# Microbiome and Metabolic Changes of Milk in Response to Dietary Supplementation With Bamboo Leaf Extract in Dairy Cows

**DOI:** 10.3389/fnut.2021.723446

**Published:** 2021-09-14

**Authors:** Zhan Jing-wei, Shen Yi-yuan, Li Xin, Zhang Hua, Niu Hui, Fang Luo-yun, Xiong Ben-hai, Tong Jin-jin, Jiang Lin-shu

**Affiliations:** ^1^Beijing Key Laboratory for Dairy Cow Nutrition, Beijing University of Agriculture, Beijing, China; ^2^State Key Laboratory of Animal Nutrition, Institute of Animal Science, Chinese Academy of Agricultural Sciences, Beijing, China

**Keywords:** microbiome, metabolomics, bamboo leaf extracts, signal pathway, dairy cows

## Abstract

Bamboo leaf extracts, with high content of flavonoids and diverse biological activities, are used in animal husbandry. Increasing evidence has suggested an association between the bovine physiology and the udder microbiome, yet whether the microbiota and the metabolites of milk affect the mammary gland health or the milk quality remains unknown. In this study, we provide a potential mechanism for the effects of bamboo leaf extracts on milk microbiota and metabolites of dairy cows. Twelve multiparous lactating Chinese Holstein dairy cows were randomly separated into two groups: basal diet as the control group (CON, *n* = 6) and a diet supplemented with 30 g/d bamboo leaf extract per head as antioxidants of bamboo leaf (AOB) group (AOB, *n* = 6) for 7 weeks (2-week adaptation, 5-week treatment). Milk samples were collected at the end of the trial (week 7) for microbiome and associated metabolic analysis by 16S ribosomal RNA (rRNA) gene sequencing and liquid chromatography-mass spectrometry (LC-MS). The results showed that the milk protein was increased (*p* < 0.0001) and somatic cell count (SCC) showed a tendency to decrease (*p* = 0.09) with AOB supplementation. The relative abundance of *Firmicutes* was significantly decreased (*p* = 0.04) while a higher relative abundance of Probacteria (*p* = 0.01) was seen in the group receiving AOB compared to the CON group. The AOB group had a significantly lower relative abundance of *Corynebacterium_1* (*p* = 0.01)*, Aerococcus* (*p* = 0.01), and *Staphylococcus* (*p* = 0.02). There were 64 different types of metabolites significantly upregulated, namely, glycerophospholipids and fatty acyls, and 15 significantly downregulated metabolites, such as moracetin, sphinganine, and lactulose in the AOB group. Metabolic pathway analysis of the different metabolites revealed that the sphingolipid signaling pathway was significantly enriched, together with glycerophospholipid metabolism, sphingolipid metabolism, and necroptosis in response to AOB supplementation. Several typical metabolites were highly correlated with specific ruminal bacteria, demonstrating a functional correlation between the milk microbiome and the associated metabolites. These insights into the complex mechanism and corresponding biological responses highlight the potential function of AOB, warranting further investigation into the regulatory role of specific pathways in the metabolism.

## Introduction

Recently, flavonoids and their analogs have been investigated as feed additives for improving milk production of dairy cows and to increase their growth and development (1, 2), because of their antibacterial and antioxidant properties (3), which have attracted wide attention. The use of antibiotics in animal feed has been banned in many countries, and therefore it is necessary to test the natural flavonoid supplements for antimicrobial activity to achieve the same benefits (4).

Bamboo leaf extracts are rich in flavonoids, with a large group of naturally occurring polyphenolic compounds existing either as free aglycones or glycosidic conjugates, based on the saturation level consisting of an A and C ring or a B-ring substitution pattern (1). These characteristic structures caused flavonoids to be targeted as key compounds with great potential in animal husbandry. Recent studies suggest that flavonoids have beneficial effects on production performance (5), ruminal bacteria (6), immune regulation, and metabolism in dairy cows (7, 8). Also, it was found that specific kinds of flavonoids from a variety of plants have different effects (9). In addition, the previous results showed that ruminal acetate and propionate, which are milk fat precursors, were significantly increased by bamboo leaf extract supplementation (7, 8). However, the influence of bamboo leaf extract on milk profiles of dairy cow has received only limited research attention. Therefore, more research needs to be done to understand the mechanism of the effects of bamboo leaf extract on the milk microbial composition and the metabolite profiles.

Mammalian colostrum and milk are important for neonates, providing not only nutrients but also complex bioactive molecules for maintaining the immune homeostasis of newborns (10, 11). Immunoregulatory compounds of milk, such as immunoglobulins, lysozyme, and lactoferrin, act as important compounds against the pathogenic and opportunistic microorganisms of mastitis to protect the udder (12, 13). Insights from recent investigations of milk microbiota (14, 15) suggest that in addition to mastitis pathogens in milk, a wide array of opportunistic and commensal microbes are found within the mammary gland (16). Recently, the commensal mammary microbes have been the focus of scrutiny by researchers comparing the microbial populations in colostrum and milk from healthy and infected parts of the udder (17, 18). Mastitis significantly changed the bacterial population, and it was seen that mastitis quarters had measurably lower numbers of proteobacteria, actinobacteria, and acidobacteria than healthy quarters, with higher numbers of Firmicutes (13). The use of antibiotics for treating mastitis and the development of drug-resistant bacteria in the milk microbiome defensive response were studied through high-throughput sequencing of bacterial 16S ribosomal RNA (rRNA) genes and bioinformatics (19). The optimal bacterial diversity of the mammary microbiota is closely associated with mammary integrity, physiology, lactation levels, milk nutrients, and resilience (20–22).

Metabolomics is widely used in studies of environmental influences and their effects on dairy cows, to elucidate the relationships among different biofluids (23), compare the composition of whole-ruminal metabolites after different dietary treatments (24), and subacute ruminal acidosis samples (25). Metabolite composition analysis can also reveal significant changes in milk quality, namely, key features affecting milk production (23) under conditions of mammary infection (13) and antimicrobial administration (26). However, little is known about the potential influence of feeding bamboo leaf extracts to dairy cows on the changes in the milk metabolites.

This study aims to investigate the mechanistic linkage between the composition of milk bacterial populations and types and concentrations of specific metabolites, and to explore the potential health benefits of bamboo leaf extract on lactation. It is expected that the use of feed additives such as bamboo leaf extract in dairy cow production can supplant the overuse of antimicrobials and the accompanying problems, and also improve herd health and milk quality.

## Materials and Methods

### Bamboo Leaf Extracts

The bamboo leaf extracts (antioxidants of bamboo leaf, AOB, ≥ 40% flavonoids) in powder form were provided by the Shanxi Sciphar Industry (Shanxi, China). The proportion of crude ash, crude protein, and water-soluble polysaccharide in AOB were 20.0, 13.0, and 15.5%, respectively (7).

### Experimental Design

The research methods were approved by the Animal Care Committee, Beijing University of Agriculture (Beijing, China) (Protocol number BUA2020021). Twelve multiparous lactating Holstein dairy cows with no clinical signs of mastitis were randomly assigned to the groups. With regard to the milk production, none of the cows showed any significant differences in yield (30.2 ± 1.4 kg/d; *p* = 0.25), days in milk (DIM, 116.2 ± 2.8 d; *p* = 0.72), parity (2.4 ± 0.6; *p* = 0.19), or body weight (BW; 596.8 ± 13.5 kg; *p* = 0.31). Of the 12 cows, six received basal feed as controls (CON) and the other six got basal feed plus supplementation with bamboo leaf extract (30 g/d per head) as the AOB group. The AOB dose used in this study was based on our previous trial (7, 8). The animals were isolated in stalls and fed *ad libitum* with unlimited water. The composition of the total mixed ration (TMR) is given in Table 1. Animals were fed and milked three times a day. The trial was continued for 7 weeks, with a 2-week adaption period and a 5-week sampling period. Milk production was recorded at each milking and feed intake was recorded over the whole trial. Milk samples were obtained on the third day of the first week and the last week of the sampling period. Individual milk samples of 50 ml with equal volumes from each udder quarter were transferred to sterile containers and held on ice. Samples were immediately taken to the lab and stored at −80°C. One of the composite milk samples (50 ml) was collected in a sample bottle with 0.6 mg/ml potassium dichromate added as a preservative and stored at 4°C for further analysis of milk components, namely, fat, protein, lactose, and SCC using an automatic ultrasonic milk composition analyzer (Bentley Instruments, MN, USA) (27).

**Table 1:** Ingredients and nutrient composition of the basal diet.

**Ingredient**	**Content, % of DM**
Alfalfa hay	6.90
Corn silage	46.32
Oat grass	2.40
Ground corn	9.88
Soybean meal	5.10
Steam-flaked corn	4.40
DDGS^a^	4.40
Corn bran	3.70
Extruded soybean	3.00
Barley	2.66
Wheat bran	2.66
Sodium cyclamate	2.40
Oats	1.50
Canola meal	1.07
Cottonseed meal	1.07
MAGALAC^b^	0.90
NaHCO_3_	0.59
Limestone	0.48
NaCl	0.27
Premix^c^	0.30
**Chemical composition, % of DM^d^**	
CP	17.4
NDF	31.1
ADF	16.6
Ether extract	5.00
Ca	0.78
P	0.44
NE_L_, Mcal/kg	1.76

a *DDGS, dried distillers grain with solubles*.

b *Church and Dwight Co., Inc., Princeton, NJ*.

c *Formulated to provide (per kg of DM) 4,560 mg of Cu, 3,000 mg of Fe, 12,100 mg of Zn, 4,590 mg of Mn, 60 mg of Co, 200 mg of Se, 270 mg of I, 10,000 IU of vitamin E, 450,000 IU of vitamin D, 2,000,000 IU of vitamin A, and 3,000 mg of nicotinic acid*.

d *Chemical composition based on chemical analysis of the total mixed ration (TMR), as described*.

### Microbial DNA Extraction, PCR, and Sequence Analysis

Bacterial DNA was isolated from milk samples with a stool DNA Kit (Omega Bio-Tek Inc., GA, USA) according to the procedure of the manufacturer. The amount and integrity of extracted DNA were assessed with a NanoDrop 1000 spectrophotometer (Nanodrop Technologies, DE, USA), and checked by agarose gel electrophoresis. DNA aliquots were stored at −80°C. The V1-V2 locus of the bacterial 16S rRNA gene was amplified using the following primer pairs: forward, 5′-CGTATCGCCT-CCCTCGCGCCATCAG-3′ and reverse, 5′-CTA-TGCGCCTTGCCAGCCCGCTCAG-3′, namely, adapters and barcode sequences using a GeneAmp 9700 (ABI, USA) as previously described (13, 18). After gel electrophoresis of amplicons, strong bands of 450 bp were excised and mixed equally as previously described (28). PCR products were isolated with a GeneJET gel extraction kit (Thermo-Fisher, MA, USA). After amplification, paired-end sequencing libraries were built by Majorbio Bio-Pharm Technology Co. Ltd. (Shanghai, China). The rRNA genes of the pooled samples were sequenced using a HiSeq system (Illumina, CA, United States) with paired-end reads of 300 base pairs by Majorbio Bio-Pharm Technology Co. Ltd (Shanghai, China).

### Analysis of Milk Microbial Composition

Bioinformatics analyses were conducted by FLASH version 1.2.11 and Quantitative Insights into Microbial Ecology (QIIME) version 1.9.1. as previously published (13). Briefly, the reads were clustered as operational taxonomic units (OTUs) by scripts of USEARCH (Version 7.1) software with a 97% similarity cut-off. The representative OTU sequences were classified into taxa with BLAST searches in the ribosomal database project (RDP) classifier (version 2.2) against the Silva (SSU123) 16S rRNA database. The selected OTUs were normalized to the relative abundance for each sample. The composition of bacterial populations was determined using the freeware Majorbio I-Sanger cloud platform (www.i-sanger.com).

Within-sample variation (alpha-diversity) was determined by ACE and Chao indices, which reflect the bacterial community abundance and Shannon and Simpson indices that show diversity. Calculations were performed on a random subset of OTUs. Between-sample microbial diversity (beta-diversity) was determined using phylogenetically based weighted-UniFrac distances (29). The OTUs were filtered to yield those with relative abundance ≥ 1% in at least one sample. Functional profiles of milk metagenomes were obtained using 16S rRNA gene sequences by PICRUSt as level III Kyoto encyclopedia of genes and genomes (KEGG) pathways (30) on the Majorbio I-Sanger cloud platform. Pathways seen in more than 10% of the samples were compared by Student's *t*-test.

### Preparation of Milk Samples for Metabolomic Analysis

The chemical composition of the milk samples was determined using the Ultimate 3000LC, Q Exactive LC-MS platform (Thermo Scientific, USA) with samples prepared according to a previous report (13). The remainder of the procedure was conducted by Majorbio Bio-Pharm Tech (Shanghai, China). In brief, methanol-acetonitrile (1:1, volume per volume) (400 μl) was added to each sample (100 μl), mixed by vortexing, and subjected to ultrasonic extraction on ice for 10 min (×3). The solution was then incubated at −20°C for 30 min and centrifuged at 13,000 g for 15 min at 4°C. Supernatants were collected, vacuum-dried, and re-dissolved in the acetonitrile-water solution (1:1, v/v) (100 μl). Then, the re-dissolvent was put into the sample holder for detection. The quality control (QC) samples consisting of all pooled milk samples were injected regularly throughout the analysis. This LC-MS-based milk metabolic profiling procedure used a 1290 UHP-LC system (Waters Corp., Milford, CT, USA) and an ethylene-bridged hybrid C18 column (2.1 mm × 100 mm id, 1.7 μm; Waters Corp., Milford, CT, USA), coupled with a triple quadrupole time of flight (TripleTOF 5600 System; AB SICEX, Framingham, MA, USA). Raw data from the metabolite fingerprinting were imported into the metabolomics processing software Progenesis QI (Waters Corp., Milford, CT, USA) for baseline filtering, peak identification, integration, correction of retention time, and peak alignment (31). The data were analyzed using XCMS (version v.3.4.4). Retention times (RT), MZ, observations (samples), and peak intensity were normalized with Excel. Differences in metabolite profiles were characterized using the https://metlin.scripps.edu/ public database, the Majorbio I-Sanger cloud platform (www.i-sanger.com), and KEGG pathway identification of the altered metabolites (www.metaboanalyst.ca/).

### Multivariate Statistical Analyses

Statistical differences were verified with Student's *t*-test (*p* < 0.05). Hierarchical clustering was performed with the Bray–Curtis similarity index by the unweighted pair-group method with arithmetic averages. The relationship of metabolite type and content to the type and abundance of bacteria in milk samples was determined from Spearman correlation coefficients and visualized using the R language (32). Statistical analyses were done using SPSS software version 21.0 (IBM, Armonk, NY). The alpha-diversity indices are shown as mean ± SD. Principal component analysis (PCA) and orthogonal partial least-squares discriminant analysis (OPLS-DA) were performed to depict changes in metabolism among the experimental groups after mean-centering and unit-variance scaling. Variables with variable importance in the projection (VIP) values > 1.0 were deemed relevant for group discrimination. The OPLS-DA model was validated by 7-fold permutation tests. Significant differences in metabolites between groups were determined using the Wilcoxon rank sum test.

### Nucleotide Sequence Accession Numbers

The raw sequences identified here were uploaded to the NCBI Sequence Read Archive (SRA; http://www.ncbi.nlm.nih.gov/Traces/sra/) under accession number SRP192494.

## Results

### Milk Production and Composition

The yield, percent fat and protein, and lactose concentration at week 5 are shown in Table 2. The protein content was higher (*p* < 0.0001) in cows with AOB compared to controls, while the milk from the AOB group also showed a lower SCC tendency (*p* = 0.09). Feeding bamboo leaf extracts did not significantly alter milk yield, but the quality was improved as the percent protein was higher in the AOB group. It is not known if changes in metabolites can be correlated with improvements in the quality of milk from AOB cows.

**Table 2:** Effects of bamboo leaf extracts on lactation performance of dairy cows.

**Items**	**CON**	**AOB**	**SEM**	***P*-value**
DMI (kg/d)	24.4	25.6	0.43	0.74
Milk production (kg/d)	30.32	32.40	1.76	0.53
4% FCM production (kg/d)^1^	34.83	41.49	1.94	<0.0001
**Milk composition**				
Fat %	4.99	5.87	0.19	0.68
Protein %	3.23	3.59	0.11	<0.0001
Lactose %	3.97	4.30	0.12	0.27
SCC^2^/ × 10^4^ cells/mL	12.59	9.88	0.66	0.09

1 *FCM, fat corrected milk*.

2 *SCC, somatic cell count*.

### Diversity and Abundance of Milk Microbiota

In total, 717,745 verified sequences from 12 samples were available for downstream analysis. The samples had a mean length of 308 base pairs with > 99% depth coverage, which indicated a data volume sufficient to accurately show alterations in most bacteria. The rarefaction curve showed that nearly all samples reached their plateaus, confirming the adequacy of the sequencing. The α-diversity indices of the bacterial communities in the two groups are given in Table 3. The differences between the two groups were non-significant, showing that feeding bamboo leaf extract did not alter the richness (ACE and Chao indices) or diversity (Shannon and Simpson indices) (*p* > 0.05) of the microbiota. These determinations may vary, however, depending on whether the calculations are based on abundance or biomass. No significant differences were found between the groups for the other α-diversity indices.

**Table 3:** Alpha diversity indices of milk bacteria.

**Item**	**CON**	**AOB**	***P*-value**
ACE	2234.02 ± 902.01	1603.32 ± 1046.49	0.29
Chao	2215.43 ± 884.95	1431.80 ± 862.85	0.15
Simpson	0.04 ± 0.02	0.17 ± 0.18	0.11
Shannon	4.93 ± 0.37	3.70 ± 1.68	0.11
Coverage	0.99 ± 0.01	1.00 ± 0.00	0.28

**Figure 1:**
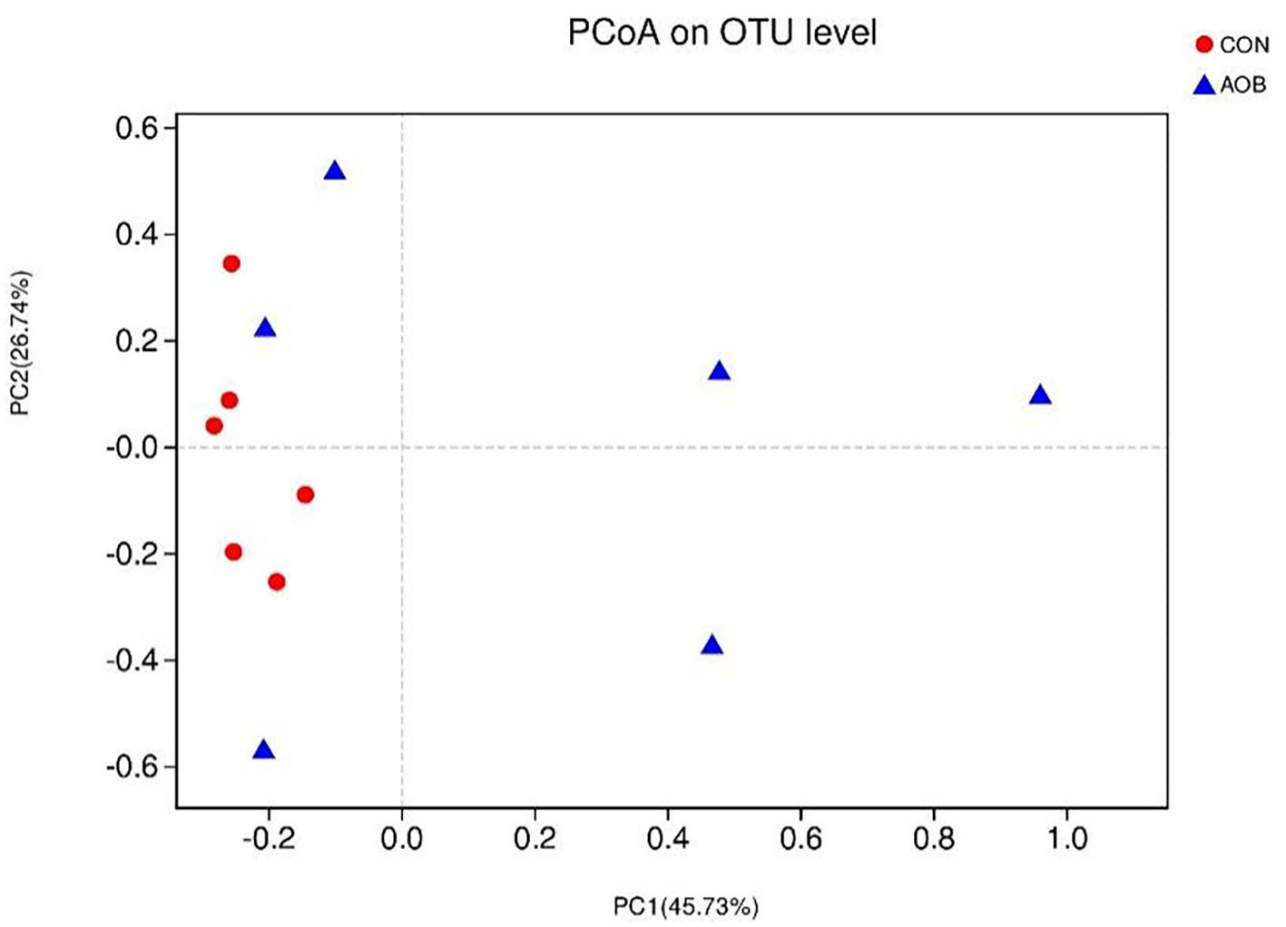
Principal coordinates analysis of bacterial communities in milk from lactating cows fed TMR with supplementation of 0 (CON) and 60 g/d of bamboo leaf extracts (AOB), *n* = 6. PCoA, principal coordinates analysis; TMR, total mixed ration.

The determination of β-diversity was made by principal coordinates analysis (PCoA) with weighted UniFrac metrics used to identify microbial distinctions between the two groups (Figure 1). PCoA revealed that principal coordinates 1 and 2 explained 45.73 and 26.74% of the total variance, respectively.

**Figure 2:**
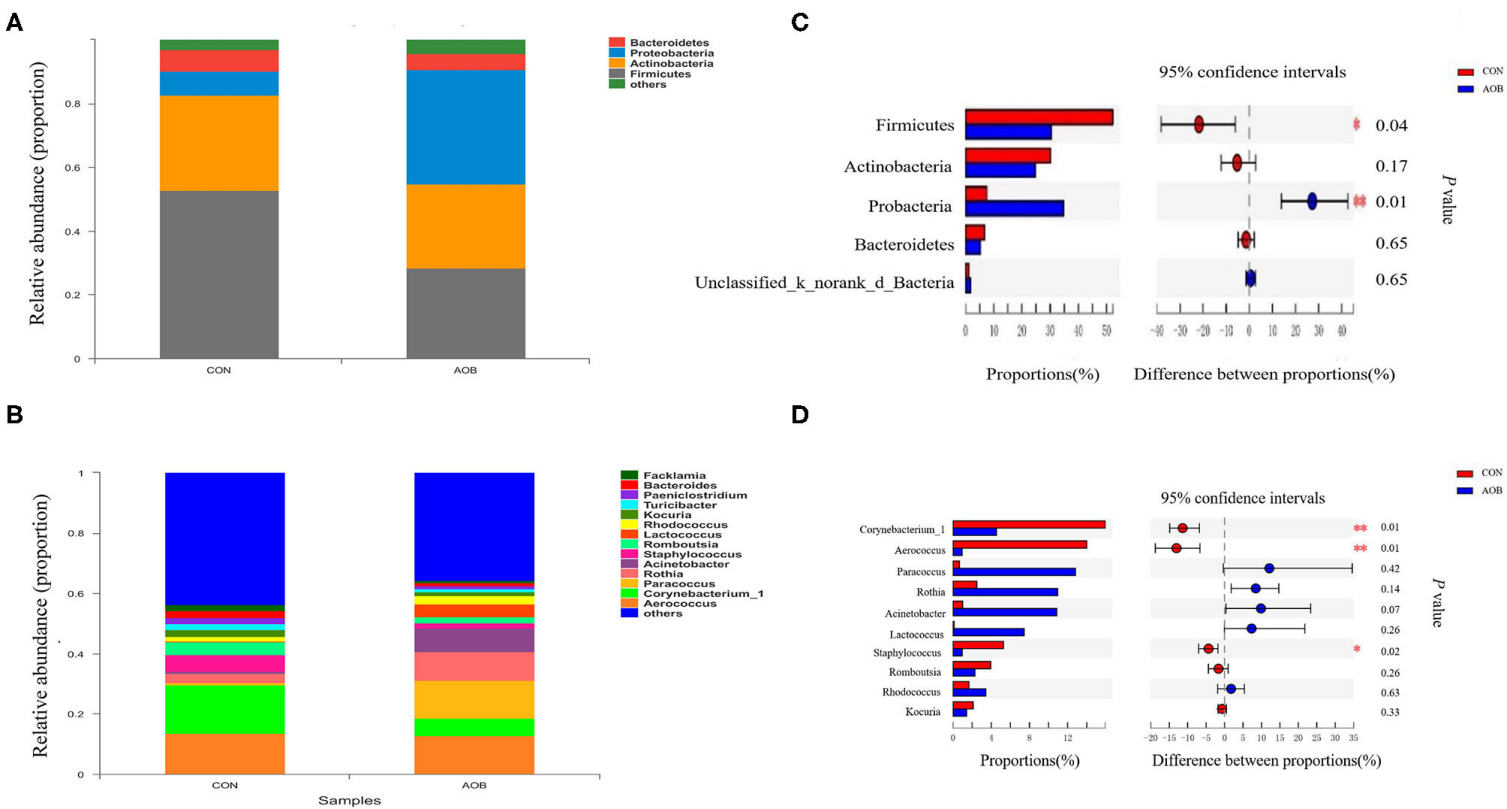
Classification of the bacterial community composition identified in milk samples from lactating cows fed TMR with supplementation of 0 (CON) and 60 g/d of bamboo leaf extracts (AOB), *n* = 6. **(A)** Phylum level. **(B)** Genus level. **(C)** Differences between the relative abundances of five predominant bacterial phyla in milk samples between two groups. **(D)** Differences between the relative abundances of the 10 predominant bacterial genera in milk samples between two groups. The extended error bar plot was generated using STAMP software. Welch's two-sided test was used, and Welch's inverted test was 0.95. TMR, total mixed ration.

Determinations of the relative abundance of taxa in the AOB and CON groups showed that the relative abundance of phylum Firmicutes was significantly decreased with AOB treatment (*p* = 0.04), while Probacteria had a relatively higher abundance (*p* = 0.01) relative to the controls (Figures 2A,C). Considering the genera, taxa with a relative abundance of ≥ 1% in at least one sample were ranked, and the top 10 most abundant with differences between the groups are shown in Figures 2B,D. Compared to the CON group, the lower relative abundance of *Corynebacterium_1* (*p* = 0.01)*, Aerococcus* (*p* = 0.01), and *Staphylococcus* (*p* = 0.02) in the AOB group was significant.

**Figure 3:**
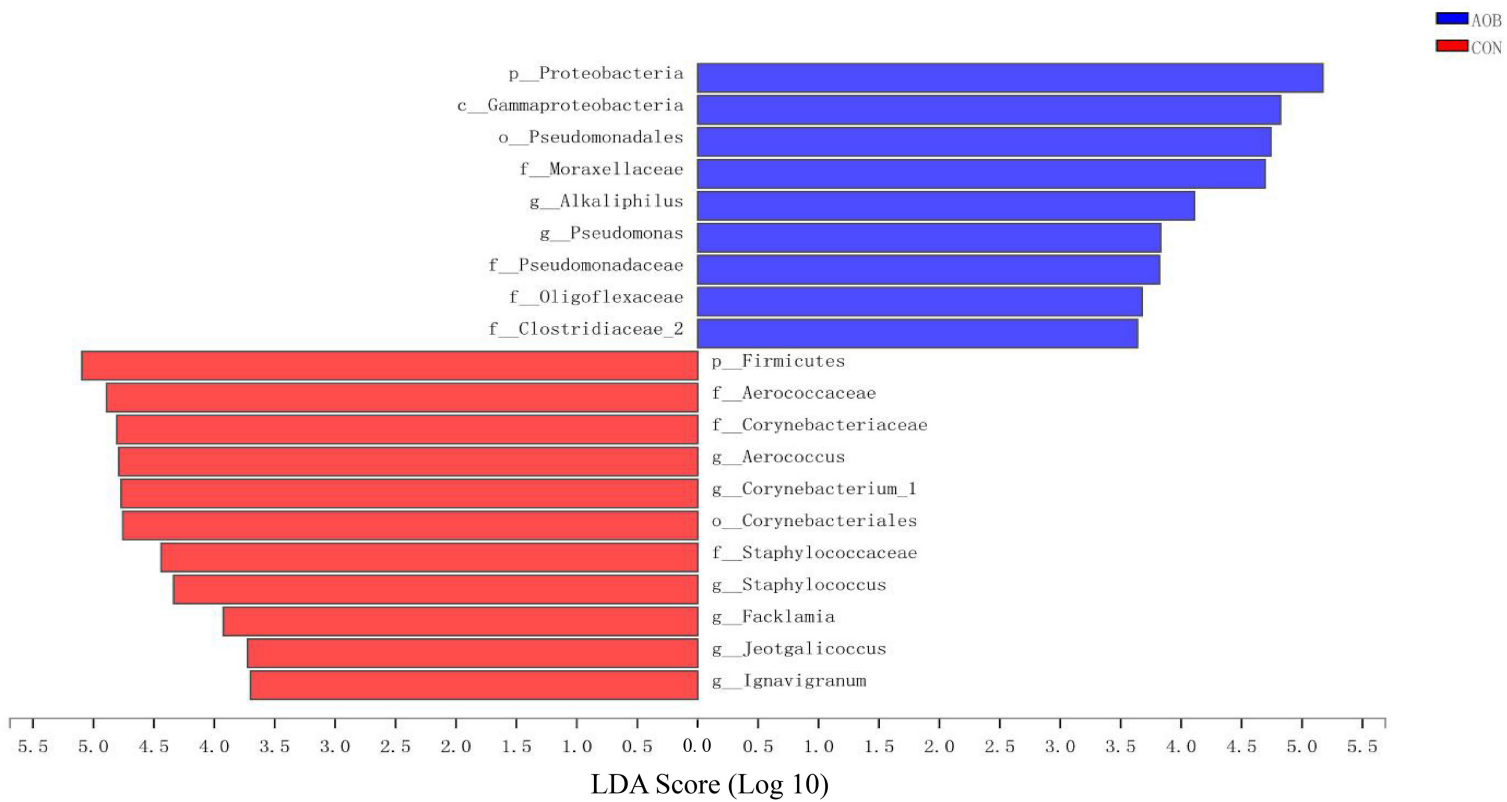
The LEfSe analysis identified the most differential abundant taxa between the two groups. Linear discriminant analysis (LDA) scores for the bacterial taxa are differentially abundant between AOB and CON groups. Only the taxa having a *p* < 0.01 and LDA > 3.5 were shown. LEfSe: linear discrimination analysis together with effect size.

Linear discrimination analysis together with effect-size analysis demonstrated significantly elevated numbers of *Aerococcus, Corynebacterium_1*, and *Staphylococcus* and a sizable reduction in *Bacteroides, Alloprevotella, Blautia*, and *Gemella* in CON relative to AOB (Figure 3).

### Microbial Functions

**Figure 4:**
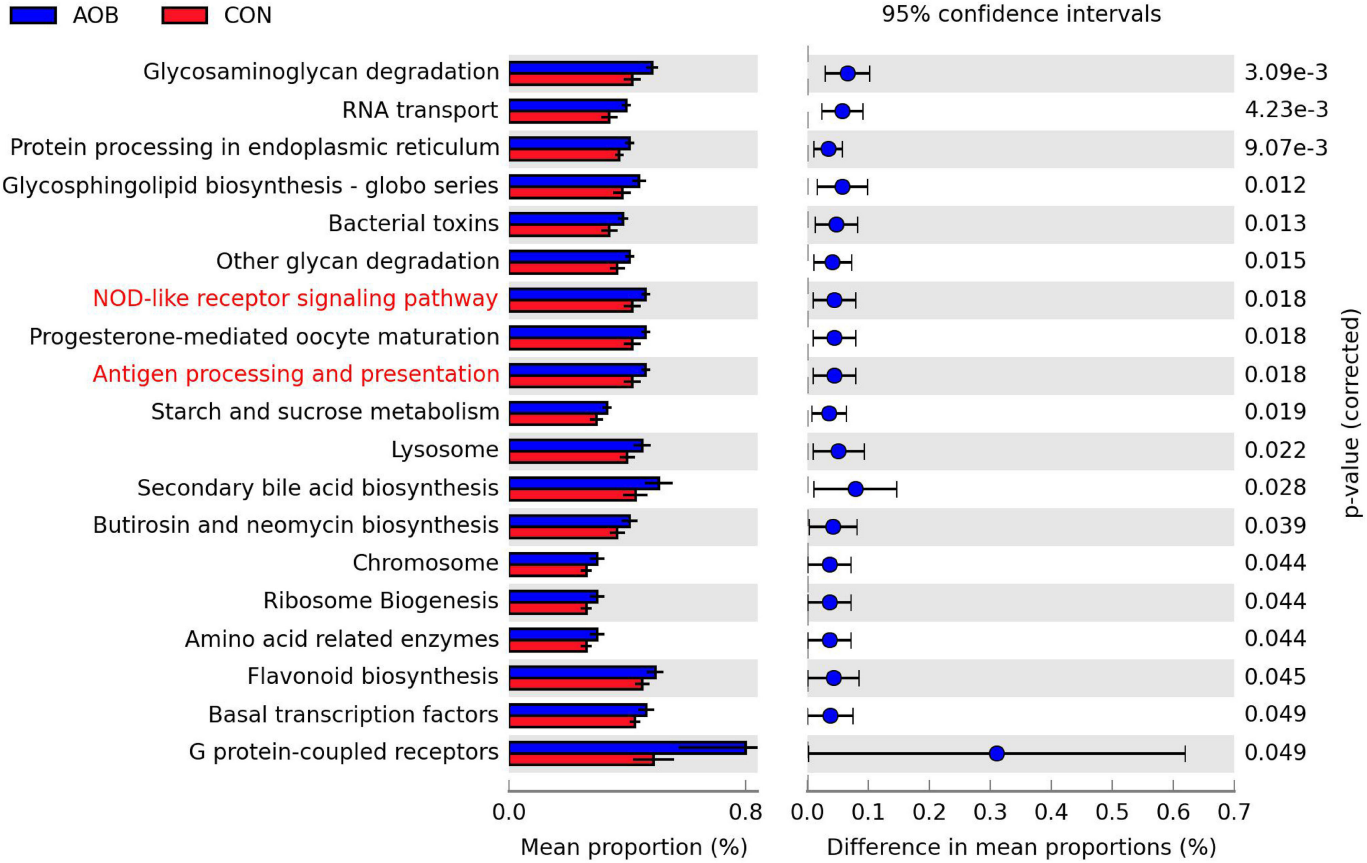
Significantly different abundance of 19 KEGG pathways (level III) of predicted microbial functional composition from 16s rRNA sequencing data with PICRUSt between two groups (*n* = 6). Highlight KEGG (Red) related to the metabolism (level I). The extended error bar plot was generated using STAMP software. Welch's two-sided test with FDR correction, *p* < 0.05. KEGG, Kyoto encyclopedia of genes and genomes.

Biological function analysis of the milk bacteria was predicted from 16S rRNA gene sequence data using PICRUSt. Identification of the relevant KEGG pathways (L-III) revealed 19 substantial category differences between the AOB group and the CON group (Student's *t*-test, *p* < 0.05; Figure 4), and eight of them that were partially related to metabolism (L-I) were significantly increased in the AOB group, namely, glycosaminoglycan degradation, glycosphingolipid biosynthesis, glucan degradation, starch and sucrose metabolism, secondary bile acid biosynthesis, butirosin and neomycin biosynthesis, amino-acid-related enzymes, and flavonoid biosynthesis. Furthermore, the NOD-like receptor signaling cascade and the antigen processing and presentation were involved in the immune functions.

### Comparison of Milk Metabolites

**Figure 5:**
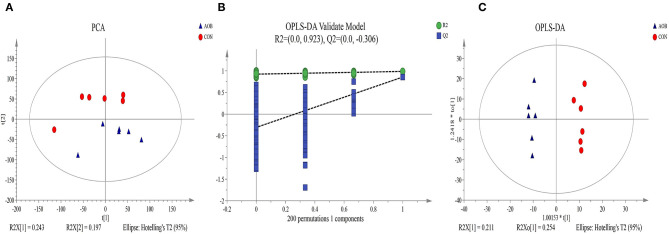
Principal component analysis (PCA) score plot **(A)**, permutation test plot **(B)**, and orthogonal partial least-square discriminant analysis (OPLS-DA) **(C)** of the CON and AOB groups based on the milk metabolites profiles. The percentage variation in the plotted principal components is marked on the axes. Each spot represents one sample, and each group of milk samples is indicated by a different color. CON, red circle; AOB, blue triangle.

A non-targeting metabolomics method was used to determine the milk metabolome after feeding with AOB. Using the statistics results and the VIP values from OPLS-DA (Table 4) 64 differentially expressed metabolites were identified in the milk samples from AOB vs. CON groups were using VIP > 1 and *p* < 0.05. They consisted mainly of glycerophospholipids, fatty acyls, prenol lipids, glycerolipids, steroids and steroid derivatives, flavonoids, and sphingolipids. Milk from cows receiving AOB contained lower levels of kojibiose, flavonoids, sphingolipids, sphinganine, and lactulose than did CON cows. PCA and OPLS-DA were performed to determine the differences in metabolite profiles between the CON and AOB groups. The PCA score plots (Figure 5A) showed that the CON group samples were distinguishable from the AOB samples. PCs one and two were responsible for 24.3 and 19.7% of the differences. The *R*^2^*Y* = 0.923 and *Q*^2^*Y* = −0.306 at a threshold <0 validated the model (Figure 5B). The OPLS-DA model quality was assessed by the validation plots. The OPLS-DA results indicated that the groups had significant differences in the metabolite composition (Figure 5C), verifying that these two analytical methods were the reliable mirrors of milk metabolite changes resulting from the AOB feeding.

**Table 4:** Comparison of metabolite contents of milk samples from lactating cows fed TMR with supplementation of 0 (CON) and 60 g/d of bamboo leaf extracts (AOB), *n* = 6.

**Metabolites**	**RT (min)**	**Ion (m/z)**	**Mass error (ppm)**	**VIP^a^**	**Fold change (AOB/CON)^b^**	***P*-value^c^**	**Tendency**
**Glycerophospholipids**							
LysoPC (14:0)	7.16	512.30	−1.99	2.91	2.09	0.01	
PC[18:1(11Z)/18:2(9Z,12Z)]	10.73	828.58	−4.95	8.67	0.86	0.01	↓
LysoPE(15:0)	6.45	484.27	−2.19	1.57	1.88	0.03	
PE(15:0/14:0)	9.89	694.47	−0.24	6.96	1.77	0.01	
LysoPC(18:0)	8.81	568.36	−1.76	8.91	1.69	0.01	
LysoPC(16:0)	7.99	540.33	−3.89	6.85	2.00	0.00	
LysoPC[20:3(5Z,8Z,11Z)]	7.83	590.35	−1.21	1.24	1.61	0.03	
1–Oleoylglycerophosphoinositol	7.93	597.30	−0.81	7.88	1.56	0.01	
PC(P−16:0/0:0)	8.38	524.34	−1.60	1.27	1.73	0.01	
PC[14:1(9Z)/14:1(9Z)]	9.58	718.47	0.56	1.10	3.08	0.00	
PE[15:0/14:1(9Z)]	9.62	692.45	0.23	2.23	2.27	0.01	
PE[22:5(4Z,7Z,10Z,13Z,16Z)/P−18:1(11Z)]	11.37	810.53	8.11	2.62	0.83	0.01	↓
PS(18:2(9Z,12Z)/18:0)	10.90	808.51	0.90	3.14	0.82	0.00	↓
PE(15:0/18:0)	10.72	750.53	0.03	4.50	0.79	0.02	↓
PE[15:0/20:3(5Z,8Z,11Z)]	10.03	772.51	0.38	1.61	1.41	0.01	
PE(14:0/14:0)	9.69	680.45	−0.51	1.88	2.48	0.00	
LysoPE(16:0)	8.23	452.28	−1.74	7.84	2.02	0.00	
LysoPE(18:0)	7.60	526.31	−2.23	1.31	1.73	0.03	
LysoPI(18:0)	8.60	601.33	−0.24	3.85	1.54	0.03	
LysoPE[18:1(9Z)]	8.33	480.31	0.20	9.54	2.32	0.00	
LysoPE(16:0)	8.24	454.29	3.01	4.67	2.32	0.00	
LysoPC(16:0)	8.14	496.34	0.23	12.41	2.58	0.00	
LysoPI[18:1(9Z)]	8.00	599.32	−0.89	2.90	1.72	0.01	
LysoPE(18:0)	8.90	482.32	0.59	5.00	1.93	0.01	
LysoPC(14:0)	7.34	468.31	0.22	2.96	2.29	0.01	
1-heptadecanoyl–sn-glycero-3-phosphocholine	8.49	510.36	0.09	1.30	2.34	0.00	
LysoPC(18:0)	8.81	524.37	0.51	7.72	2.30	0.00	
LysoPE(14:0)	7.44	426.26	−0.23	1.09	2.98	0.00	
PS[18:0/18:2(9Z,12Z)]	7.03	752.51	−9.59	1.28	3.46	0.04	
LysoPC(15:0)	7.76	482.32	−0.11	1.58	2.61	0.00	
**Fatty acyls**							
cis-9,10-Epoxystearic acid	7.40	297.24	−2.44	5.31	2.42	0.02	
Kojibiose	0.59	377.08	0.24	24.53	0.81	0.00	↓
5-Hexyltetrahydro-2-furanoctanoic acid	7.64	343.25	−2.83	1.28	2.33	0.03	
Rollinecin A	9.72	659.46	−2.08	1.92	2.49	0.01	
4-Deoxyannoreticuin	9.78	625.47	−2.40	1.16	2.79	0.01	
11,14,17-Eicosatrienoic acid	8.84	339.29	0.57	4.44	2.72	0.02	
Erythro−6,8-tricosanediol	9.27	401.34	0.03	1.86	1.49	0.03	
Linoleamide	8.28	280.26	−0.37	1.10	1.32	0.00	
**Prenol lipids**							
Crocin	8.91	975.37	−5.04	2.05	0.76	0.02	↓
26-Methyl nigranoate	9.25	505.33	−1.70	3.84	2.60	0.00	
(R)-6′-O-(4-Geranyloxy-2-hydroxycinnamoyl)-marmin	7.93	665.29	4.97	1.49	1.42	0.04	
7C-aglycone	2.02	595.23	1.87	1.01	3.94	0.00	
13′-Hydroxy-gamma-tocopherol	10.12	474.42	−0.16	2.16	1.31	0.05	
3-Hydroxy-10′-apo-b,y-carotenal	9.39	434.31	0.09	1.05	0.57	0.03	↓
**Glycerolipids**							
DG(18:3(9Z,12Z,15Z)/22:0/0:0)	10.44	719.59	4.16	1.03	0.82	0.01	↓
MG(0:0/14:0/0:0)	8.08	285.24	−0.34	2.70	2.39	0.01	
DG(12:0/12:0/0:0)[iso2]	10.10	479.37	−0.28	3.90	1.37	0.02	
MG(0:0/18:0/0:0)	9.33	341.31	0.06	1.95	2.42	0.02	
**Steroids and steroid derivatives**							
Pregnanediol	8.82	685.54	0.16	1.54	7.17	0.00	
Calusterone	9.71	677.48	4.21	1.30	2.48	0.00	
Campesteryl p-coumarate	9.18	545.40	6.35	1.10	2.73	0.00	
O-methoxycatechol-O-sulfate	2.48	203.00	−0.97	3.82	2.62	0.04	
**Flavonoids**							
2″-(6″-p-Coumaroylglucosyl)quercitrin	0.80	777.16	−4.81	7.50	0.44	0.01	↓
Moracetin	0.95	787.19	−4.75	7.87	0.64	0.00	↓
Brassicoside	0.93	839.16	−5.40	3.10	0.50	0.01	↓
**Sphingolipids**							
SM(d18:1/12:0)	9.93	691.50	−0.48	3.14	1.54	0.00	
N,N-dimethyl-safingol	7.59	330.34	0.11	1.29	0.44	0.00	↓
C16 sphinganine	5.91	274.27	−0.22	3.61	0.54	0.00	↓
**Benzene and substituted derivatives**							
Gentisic acid	2.60	153.02	2.46	2.67	3.10	0.00	
Hippuric acid	2.37	180.07	−1.45	3.44	1.52	0.04	
**Organonitrogen compounds**							
Sphinganine	6.80	302.31	0.20	2.09	0.49	0.00	↓
**Organooxygen compounds**							
Lactulose	0.60	365.11	0.47	20.11	0.69	0.01	↓
**Pyrans**							
Ethyl maltol	2.37	105.03	1.83	1.79	1.44	0.03	
**Carboxylic acids and derivatives**							
L-pyridosine	2.01	255.13	−0.83	1.39	3.12	0.00	

a *VIP: variable importance in the projection obtained from the OPLS model with a cut-off of 1.5*.

b *FC: fold change, the ratio of the mean value of the peak area obtained from the AOB group and the mean value of the peak area obtained from the CON group. An FC <1 means that the metabolite is lower in milk from AOB cows than in milk from CON cows*.

c *p-values from nonparametric Wilcoxon-Mann-Whitney test*.

**Figure 6:**
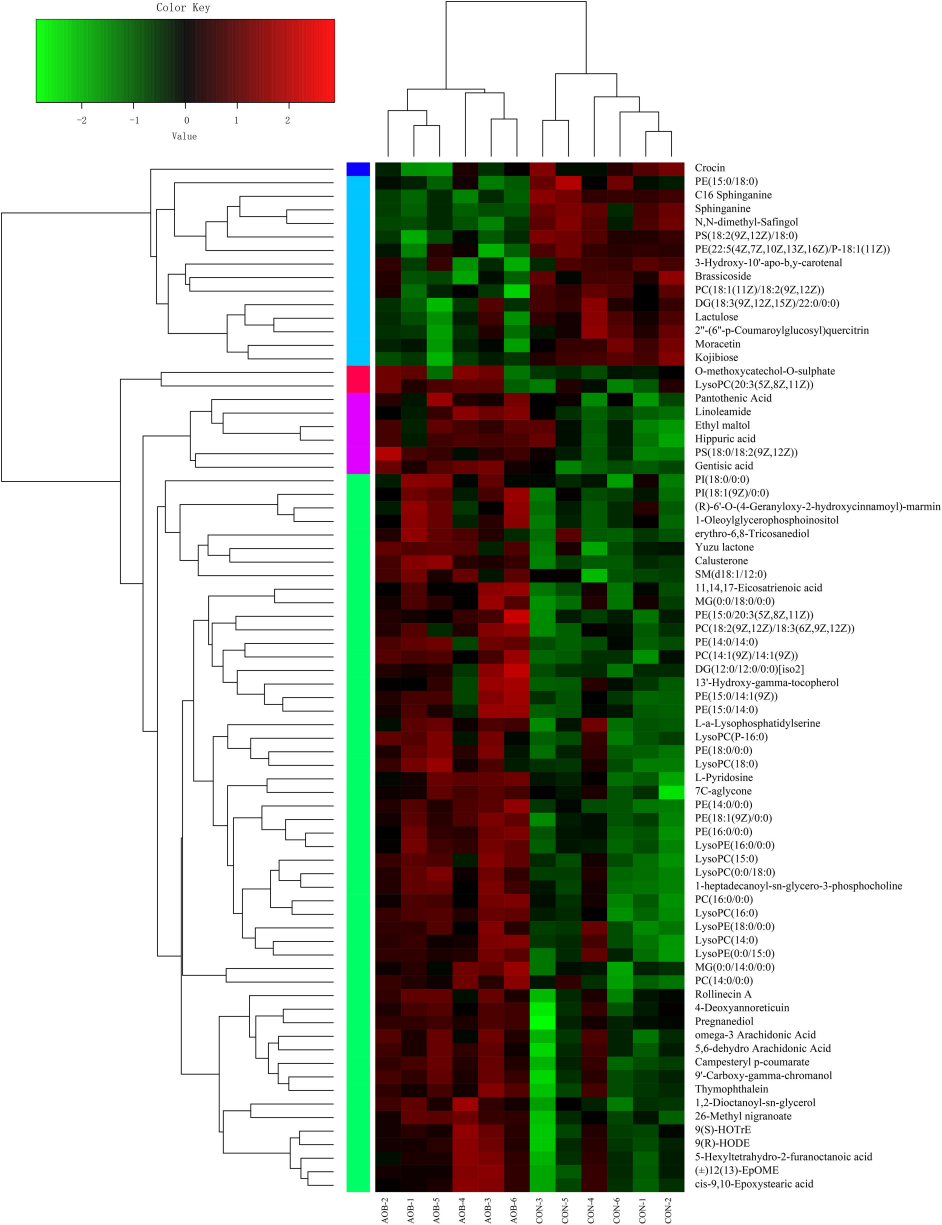
Hierarchical clustering analysis (HCA) and heat map for different milk metabolites from the CON and AOB groups. The heatmap shows the significantly changed metabolites between the two groups. Columns in the heatmap represent one sample, and rows represent one metabolite. The color bar showing green to red indicates the relative content of the metabolites (*n* = 6).

**Figure 7:**
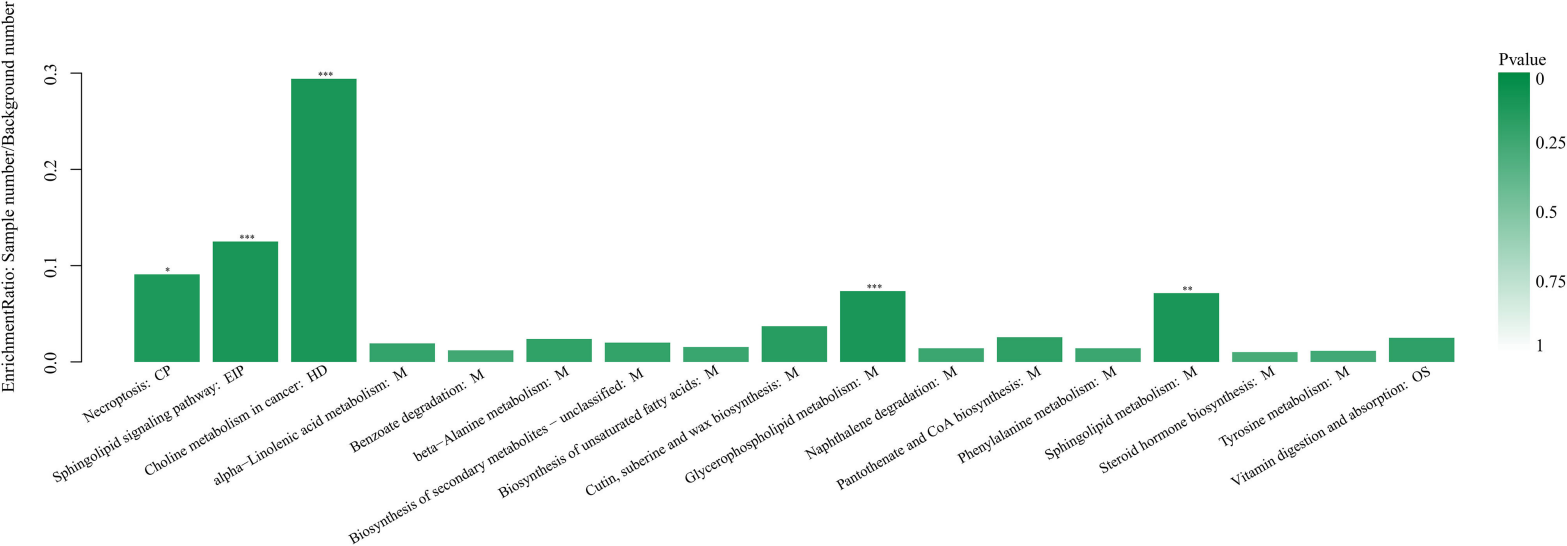
Kyoto encyclopedia of genes and genomes pathway enrichment analysis based on the changed metabolites between the CON and AOB groups. *p < 0.05, **p < 0.01, ***p < 0.001; Fisher's exact test with BH correction. KEGG, Kyoto encyclopedia of genes and genomes.

Hierarchical clustering analysis (HCA) with a heat map was performed to illustrate the differences in metabolite levels because of the AOB supplementation, and these were easily distinguished from those of the CON group (Figure 6). Glycerophospholipids and fatty acyls were upregulated, while the AOB-downregulated metabolites included moracetin, sphinganine, and lactulose. Enrichment analysis showed that metabolic pathways, such as sphingolipid signaling, glycerophospholipids metabolism, sphingolipid metabolism, and necroptosis were significantly affected in response to AOB supplementation (Figure 7).

### Relationship Between Milk Microbial Communities and Metabolite Production

**Figure 8:**
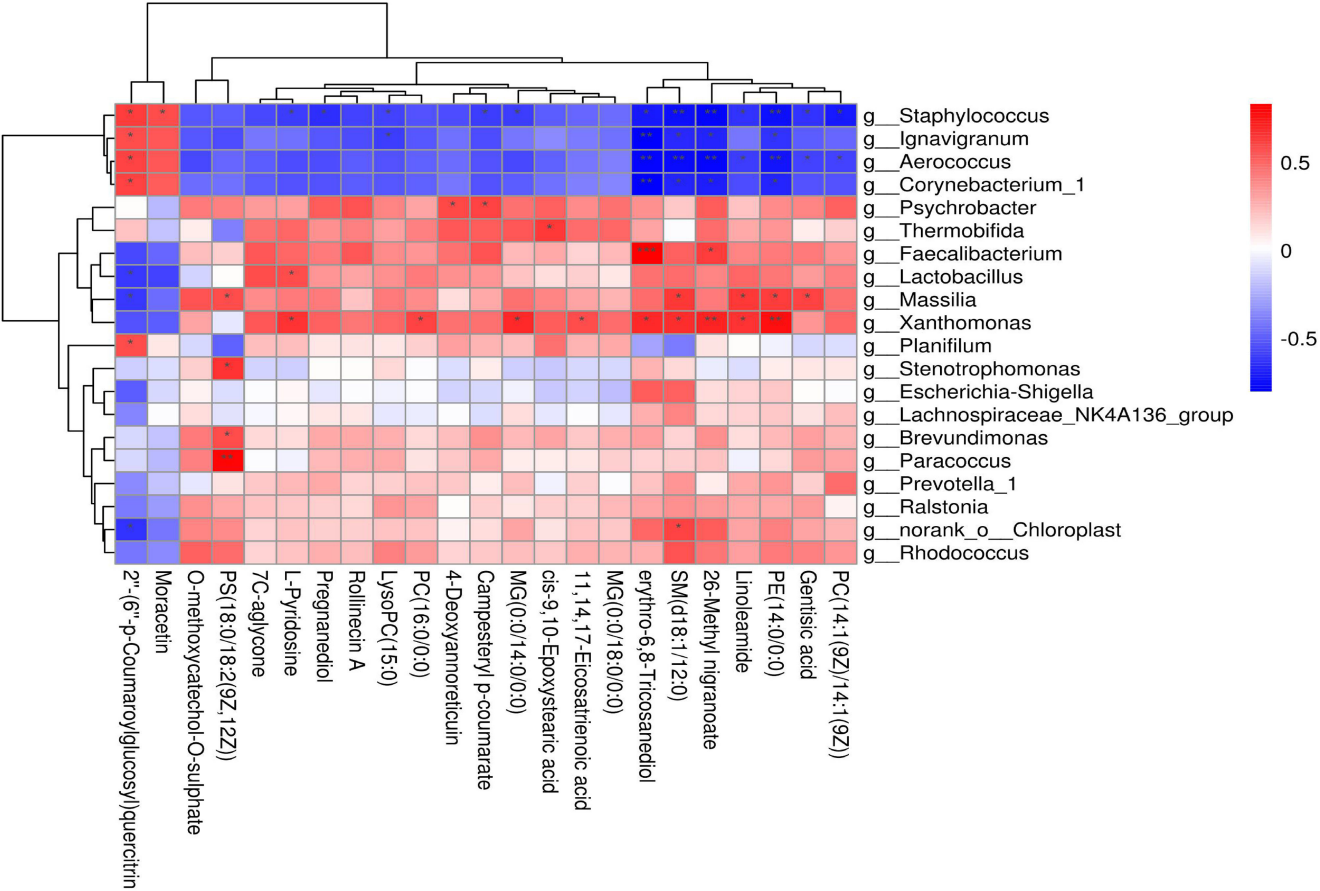
Correlation analyses between milk bacteria genus and altered metabolite levels in the CON and AOB groups. Each row in the graph represents a genus, each column represents a metabolite, and each lattice represents a Pearson correlation coefficient between bacteria and metabolites. Red represents a positive correlation, while blue represents a negative correlation (*0.01 < *p* < 0.05; **0.001 < *p* ≤ 0.01; ***p < 0.001).

Based on Spearman's correlation coefficients clear correlations were observed between the significantly affected milk microbiota (at the genera level) and metabolite composition (0.5 < *r* < −0.5, *p* < 0.05). Comparison of the metabolite profiles in the two groups showed significant differences with a strong correlation to the type of bacteria (Figure 8). Relative abundances of *Staphylococcus, Ignavigranum, Aerococcus, Corynebacterium_1, Massilia*, and *Xanthomonas* were strongly related to levels of specific metabolites (Figure 8, top). *Staphylococcus* was positively correlated with moracetin but negative for pregnanediol. The significantly decreased metabolite PS[18:0/18:2(9Z,12Z)] was positively correlated with *Paracoccus, Brevundimonas, Stenotrophomonas*, and *Massilia*, while SM (d18:1/12:0) was associated with most of the bacterial genera identified. Gentisic acid was negatively correlated with *Staphylococcus* and *Aerococcus* but positively correlated with *Massilia*. Overall, these results indicated that in response to dietary AOB supplementation, milk microbiota was associated with significant changes in metabolite levels, especially those related to the sphingolipid metabolism.

## Discussion

We have shown that bamboo leaf extract moderately increased milk protein and decreased SCC tendency. Although we did not observe any changes in the milk microbiota diversity in the AOB group at the phylum level, the numbers of Firmicutes significantly decreased and the relative abundance of Probacteria was significantly increased relative to the CON group. The well-recognized functional data on milk bacterial communities showed that their richness and diversity were strongly dependent on the health of the animals (26, 33, 34). Thus, we propose that AOB feeding affected the milk microbiota of the animals, which in turn resulted in the observed changes in the milk protein levels.

The metabolites identified in the AOB group were strongly associated with the sphingolipid signaling pathway, glycerophospholipid metabolism, and sphingolipid metabolism. Previous studies demonstrated that sphingolipid metabolites could act as signaling molecules to modulate a range of functions related to immune responses and inflammation (35). This might be associated with the AOB-mediated decrease in SCC. Interestingly, many of the metabolites detected were significantly associated with potential pathogens, such as *Staphylococcus, Massilia*, and *Aerococcus*. Taken together, the findings demonstrate the power of analyzing the correlations between the microbiota of an animal and identifiable metabolic changes in testing feed additives such as bamboo leaf extract for their effects on improving animal health, milk production, and quality.

The mechanistic insights into how AOB alters the milk microbiota were strengthened by the findings obtained in this study. First, milk microbiota composition was significantly shifted at the phylum and genera level in the AOB group. Plant flavonoids have been reported to have anti-inflammatory activity and antimicrobial properties in dairy cows (36–38), specifically through their influence on the rumen or gut microbiota (4, 39, 40). The study of the effect of AOB showed similarities in terms of the relative abundance of Firmicutes, which was significantly decreased, while Probacteria was elevated. These species are also found in the rumen microbiome (41, 42), and we hypothesize that microbial transfer between the rumen and the udder occurred. Recent reports provide evidence for an enteromammary pathway by means of the blood or lymph in humans and mice (43, 44), and the passage from mothers to neonates from gut to breast milk (45). We also found that the relative abundance of *Corynebacterium_1, Aerococcus*, and *Staphylococcus* were decreased by the AOB treatment. These are the most frequently seen genera in the milking area and are considered to be the leading candidates for IMI (46–48). It is well-known that physiological changes affect milk production and quality, udder health, and dairy sustainability. In addition, the milk microbiota and metabolites can be affected by many factors, such as dynamic physical, chemical, and predatory environments (49, 50). These mechanistic insights into the responses of milk microbiota to AOB support its potential as a candidate prophylactic or therapeutic feed additive. The antibacterial activity of AOB also makes it a possible replacement for the large-scale overuse of antimicrobials in the dairy industry. The specific correlations between the effects of AOB on the udder microbiota and the presence of certain metabolites have important implications for understanding how the milk microbiota influences udder health and susceptibility to mastitis.

Current research has highlighted the close connection between the milk bacterial composition and SCC in dairy cows (18, 48). This suggests a significant contribution of these microbes for maintaining homeostasis in the udder ecosystem to protect the mammary gland from pathogens (51, 52). In agreement with this data, we showed that SCC was significantly decreased by AOB along with the number of pathogens such as *Staphylococcus*. Previous studies have suggested that flavonoids decreased SCCs in milk from dairy cows (4, 53). However, investigations of the use of flavonoids extracted from the bamboo leaf in dairy cows are limited. The previous study supported AOB as a potentially important compound for treating subclinical mastitis and mild-lactation in dairy cows (7, 8, 54). Accompanied by a decrease in the level of inflammatory factors and the SCC, the findings shed new light on the benefits of flavonoid supplementation in the ruminant feed. How the milk microbiota is involved in triggering inflammatory and immune regulation, leading to a decrease in SCC is a subject that still needs further investigation.

In this study, 64 differentially expressed milk metabolites were identified in milk sampled from cows receiving AOB-supplemented feed compared to controls given only the usual feed. The data were classified according to the human metabolome database (HMDB) and the KEGG. The most significant changes in milk metabolites from AOB treatment occurred for glycerophospholipids, particularly those in the sphingolipid metabolic pathways (Table 4, Figure 7). Previous studies suggested that metabolomics could be utilized to provide important insights into the physiological and biochemical status of an organism (55). Hence, these findings highlight the role of AOB function in response to metabolites. We showed that glycerophospholipids and fatty acyls were significantly upregulated in the AOB group compared with the CON group. In contrast, 15 metabolites were significantly downregulated in the AOB group, namely, moracetin, sphinganine, and lactulose. Previous studies demonstrated that flavonoids as bioactive compounds in milk play an important role in antioxidant defense (56). HPLC analysis of specific types of forage fed to cows in comparison to the metabolite content of their milk suggested that flavonoids affected the quality and sensory traits of the milk and milk products (57, 58). Therefore, the data indicate that glycerophospholipids and fatty acyls could be potential biomarkers in milk for gauging the response to feed supplementation with flavonoid-containing plant extracts such as AOB.

In addition, it has been demonstrated that alterations in the microbiota can affect the systemic metabolite composition (59). In this previous study, we proved that dysbiosis of the milk microbiota of cows with subclinical *Streptococcus agalactiae* mastitis was significantly correlated with some metabolic biomarkers. Not only did we identify differences in the metabolite profiles of milk from cows with subclinical *S. agalactiae* mastitis, but also pinpointed the likely pathways that generated these metabolite differences (13). Furthermore, the milk microbiota of primiparous cows with subacute ruminal acidosis, which is a metabolic disorder attributed to dysbiosis of the rumen and hindgut microbiota, were found to include pathogenic and opportunistic bacteria, such as *Stenotrophomonas, Streptococcus, Pseudomonas*, and *Alcaligenes* (60). Consistent with this evidence, changes in the bacterial population types and abundance in the milk can not only affect the udder health and physiology, but also have adverse effects on the milk quality, production, dairy sustainability, and mastitis susceptibility (20–22). These consistent associations support the hypothesis that changes in milk microbial communities may be related to changes in the milk composition or the metabolic processes involved in milk synthesis, specifically through dietary changes, such as AOB feed additives.

One especially intriguing observation from this study was that gentisic acid was found to be significantly elevated (3.10-fold) in the AOB group compared with the control group (CON). It had previously been demonstrated that gentisic acid is a by-product of tyrosine and benzoate metabolism and exhibits antioxidant, antirheumatic, anti-inflammatory, and antibiotic activities (61, 62), which might help to explain the role of AOB in decreasing SCC in milk and altering the microbiota. Quercetin is a flavonoid that affects angiogenesis and vascular inflammation in ruminants (37). Its anti-inflammatory mechanism was studied by administering multiple intramammary treatments to dairy cows with clinical mastitis resulting in a decrease in SCC (53). In the present study, quercetin was decreased by AOB supplementation, yet a significant positive correlation was found between quercetin and the presence of *Corynebacterium_1, Aerococcus*, and *Staphylococcus*. It is well-known that pregnanediol promotes the development of the mammary gland lobule and acinus based on the estrogen assist (63). In this study, we showed that pregnanediol was significantly increased by 7.17-fold in the AOB group, which supports the idea that AOB can stimulate milk production, and also account for the negative correlation between pregnanediol and *Staphylococcus*. Additionally, bovine lymphocyte antigen (BoLA) gene polymorphism effects on the milk microbiota directly or indirectly regulated the mammary gland immune system and may be important in the future development of novel strategies for preventing or treating mastitis (64).

Although scientific evidence for the *in vivo* efficacy of bamboo leaf extract in dairy cows is limited, the evidence so far supports its health-promoting properties. This study does have some limitations, however. Milk was only sampled during the first and last weeks of the sampling period. If samples were collected every week and the correlation index was calculated, the time at which bamboo leaf extract had the strongest effect and the changes to the milk microbiota at that time could be explored. Also, the effect of AOB on immune regulation warrants further study. Although the correlation between the changes to the microbiota and the content of metabolite biomarkers has been studied, further exploration will be required to understand the mechanism linking the alterations of the microbiota and metabolites to the bamboo leaf extract treatment. In this previous study, we found relatively low bioavailability of AOB administered in the rumen because of degradation by ruminal bacteria. It is not known if AOB would be absorbed through the small intestine in ruminants if degradation was blocked. In addition, the major bioactive elements in bamboo leaf extract need to be identified by their chromatographic fingerprints.

## Conclusions

Taken together, the data indicate that AOB supplementation increased milk protein and decreased SCC, and also changing the structures of bacterial communities in milk. Moreover, AOB could decrease the relative abundance of *Corynebacterium_1, Aerococcus*, and *Staphylococcus*. The results also revealed substantial modifications in the metabolites associated with AOB supplementation and correlated with integrated pathway analyses, suggesting that some of the 64 different types of metabolites identified in the milk may be biomarkers for the AOB activity. The results from the milk metabolic pathway analysis are promising for the investigation of the antimicrobial and anti-inflammatory properties of the bamboo leaf extracts that are closely associated with the metabolic pathways for glycerophospholipid and sphingolipid metabolism. The complexity of the mechanisms involving immune regulation and associated metabolic pathways provides a great incentive for exploring the use of AOB as a feed additive to improve the mammary gland health of dairy cows.

## Data Availability Statement

All raw sequences were submitted to the NCBI Sequence Read Archive (SRA; http://www.ncbi.nlm.nih.gov/Traces/sra/) under accession number SRP192494.

## Ethics Statement

The animal study was reviewed and approved by the Animal Care Committee, Beijing University of Agriculture (Beijing, China).

## Author Contributions

TJ-j, JL-s, and ZH contributed to conception and design of the study. FL-y and ZJ-w performed the statistical analysis. ZJ-w, SY-y, and LX wrote the first draft of the manuscript. SY-y, NH, and LX wrote sections of the manuscript. All authors contributed to manuscript revision, read, and approved the submitted version.

## Funding

This study was financially supported by the Project of the National Natural Science Foundation of China (Grant No. 31802091) and the Key Project of the Beijing Municipal Education Commission (20JF0008).

## Conflict of Interest

The authors declare that the research was conducted in the absence of any commercial or financial relationships that could be construed as a potential conflict of interest.

## Publisher's Note

All claims expressed in this article are solely those of the authors and do not necessarily represent those of their affiliated organizations, or those of the publisher, the editors and the reviewers. Any product that may be evaluated in this article, or claim that may be made by its manufacturer, is not guaranteed or endorsed by the publisher.
